# Quantification of serum *C*-mannosyl tryptophan by novel assay to evaluate renal function and vascular complications in patients with type 2 diabetes

**DOI:** 10.1038/s41598-021-81479-y

**Published:** 2021-01-21

**Authors:** Shuhei Morita, Yoko Inai, Shiho Minakata, Shohei Kishimoto, Shino Manabe, Naoyuki Iwahashi, Kazuhiko Ino, Yukishige Ito, Takashi Akamizu, Yoshito Ihara

**Affiliations:** 1 First Department of Medicine, Wakayama Medical University, 811-1 Kimiidera, Wakayama, Wakayama 641-0012 Japan; 2 Department of Biochemistry, Wakayama Medical University, 811-1 Kimiidera, Wakayama, Wakayama 641-0012 Japan; 3 Pharmaceutical Department & The Institute of Medicinal Chemistry, Hoshi University, 2-4-41 Ebara, Shinagawa, Tokyo 142-8501 Japan; 4 Research Center for Pharmaceutical Development, Graduate School of Pharmaceutical Sciences & Faculty of Pharmaceutical Sciences, Tohoku University, 6-3 Aoba, Sendai, Miyagi 980-8578 Japan; 5 Department of Obstetrics and Gynecology, Wakayama Medical University, 811-1 Kimiidera, Wakayama, Wakayama 641-0012 Japan; 6 RIKEN Cluster for Pioneering Research, 2-1 Hirosawa, Wako, Saitama 351-0198 Japan; 7 Department of Chemistry, Graduate School of Science, Osaka University, 1-1 Machikaneyama, Toyonaka, Osaka 560-0043 Japan

**Keywords:** Endocrinology, Nephrology

## Abstract

*C*-Mannosyl tryptophan (CMW) is a unique glycosylated amino acid, and a candidate novel biomarker of renal function. In type 2 diabetes (T2D), a combination of metabolites including CMW has recently been the focus of novel biomarkers for the evaluation of renal function and prediction of its decline. However, previous quantification methods for serum CMW have several limitations. We recently established a novel assay for quantifying serum CMW. Serum CMW from 99 Japanese patients with T2D was quantified by this assay using hydrophilic interaction liquid chromatography. The serum CMW levels were cross-sectionally characterized in relation to clinical features, including renal function and vascular complications. Serum CMW level was more strongly correlated with serum creatinine and cystatin C levels and with eGFR than with albumin urea level. The ROC curve to detect eGFR < 60 ml/min/1.73 m^2^ revealed that the cutoff serum CMW level was 337.5 nM (AUC 0.883). Serum CMW levels were higher in patients with a history of macroangiopathy than in those without history. They correlated with ankle-brachial pressure index, whereas cystatin C did not. Serum CMW levels quantified by the novel assay could be useful in evaluation of glomerular filtration of renal function and peripheral arterial disease in T2D.

## Introduction

*C*-Mannosyl tryptophan (CMW) is a unique glycosylated amino acid, which is composed by linking a monosaccharide, mannose and an aromatic amino acid, Trp, through a C–C bond^1^. CMW was first isolated from human urine^2^, its structure confirmed later^3^. The structure was also identified in human ribonuclease 2 as a post-translational modification of protein^4^. CMW was revealed to be produced as a unique structure in proteins containing the consensus amino acid sequence Trp–X–X–Trp/Cys (W–X–X–W/C), in which the first Trp is *C*-mannosylated by a specific *C*-mannosyltransferase with dolichyl-P-mannose as a mannose donor^1,5,6^. *C*-Mannosyltransferase is encoded in *dpy-19* gene in *Caenorhabditis elegans*^7^, and its orthologues, *DPY19L1* and *DPY19L3* have been identified in mammals^8,9^. The consensus W–X–X–W/C sequence is frequently *C*-mannosylated in proteins of the thrombospondin type 1 repeat (TSR) superfamily (e.g., thrombospondin, F-spondin, R-spondin, ADAMTS-like protein 1, and complements) and type I cytokine receptor family (e.g., erythropoietin (EPO) receptor, thrombopoietin (TPO) receptor, and IL-21 receptor), and others (e.g., ribonuclease 2, mucins, and hyaluronidase 1)^5,6^. Regarding free monomeric CMW in cells, the mechanism of CMW synthesis has not yet been clarified, and it may be generated by the proteolytic degradation of *C*-mannosylated proteins in cells or alternatively synthesized by binding a free Trp to a mannose. We recently found that CMW is in part produced through autophagic pathways in cells under the condition of nutritional starvation^10^. Further investigation is required, however, to determine how the levels of CMW are differentially maintained and controlled in each tissue, body fluid, and cell type in the body.

Diabetic kidney disease (DKD) develops in approximately 40% of patients with diabetes and is the leading cause of chronic kidney disease (CKD)^11^. In daily clinical practice, the estimated glomerular filtration rate (eGFR), based on the levels of serum creatinine or, more recently cystatin C, is widely employed to evaluate renal function^12^. In addition to albuminuria, quantification of eGFRs based on those two markers is strongly recommended in several guidelines for assessment of renal function in relation to diabetic nephropathy, which is necessary to confirm the stage of CKD^13,14^. However, these biomarkers still have several limitations^12,15^. Serum creatinine level is affected by muscle mass, for example, so evaluation of eGFR on the basis of serum creatinine levels in subjects with decreased muscle mass must be made cautiously; this is especially often observed in elderly people. Similarly, although cystatin C level shows improved accuracy for the early detection of CKD over serum creatinine level, it can be affected by several factors, such as inflammation and smoking.

Recent studies have shown that several metabolites could be candidate novel biomarkers for evaluating renal function^12,16–18^. Although various studies have shown promising results using a combination of metabolites to assess renal function, the value of a single metabolite has not yet been fully investigated. Among candidates, CMW was one of the metabolites found to have especially high potential in previous studies^16,18–21^. However, there are remaining issues concerning CMW. Measurement of serum CMW by reverse-phase HPLC could be affected by impurities in the serum, so reports with accurate evaluation of serum CMW are likely limited^20^. Moreover, although CMW is a candidate renal function marker for CKD, there are still limited reports regarding which renal dysfunction factors, such as glomerular filtration or excretion, could be related to CMW level, especially in DKD. Furthermore, in DKD, it is unclear whether dysregulated glucose metabolism or other clinical factors could be related to serum CMW level.

Here, we first quantified serum CMW levels by the novel assay and then characterized them in relation to the clinical characteristics including present biomarkers for renal function in patients with type 2 diabetes (T2D). Next, we investigated the clinical advantage of quantifying the absolute value of CMW beyond the assessment of renal function.

## Results

### Measurement of serum CMW

Serum CMW was detected and measured by fluorescence intensity using ultra performance liquid chromatography (UPLC), as described in the “Materials and methods” section. Serum samples from control subjects and patients were separated by hydrophilic interaction liquid chromatography (HILIC)^22^. CMW is an endogenous substance in humans, but the validity of the assay method with fluorescence measurement was assessed in accordance with guidelines on bioanalytical method validation^23^. The linearity of the assay was examined in accordance with our previous report^22^, and the slope of calibration curve was 2.04 × 10^–4^ ± 8.92 × 10^–6^ and the correlation coefficient (r^2^) was at least 0.99. In terms of CMW sensitivity, limit of detection (LOD) and limit of quantification (LOQ) were determined as 1 nM and 5 nM, respectively. The specificity of the method was examined by analyzing the separation of CMW from presumed isomers, such as *N*-mannosyl Trp and *C*-glucosyl Trp^10^. CMW was confirmed to be distinctively detected with HILIC in this assay, indicating sufficient selectivity of the assay method. Although analyte-free serum was not obtained in the present study, we examined the extraction recovery and matrix effect. The recovery and matrix effect were 88.17–105.26% and 100.12–117.47%, respectively (Supplementary Table 1). The overall relative standard deviation (RSD) calculated for CMW in quality control (QC) concentrations were < 20%. To assess the precision and accuracy of the assay, serum samples were divided into two groups: those with low (< 300 nM) and those with high (≧ 300 nM) concentrations of CMW. In this assay, the CMW concentration, which is the sum of endogenous and spiked CMW in spiked serum samples, was robustly measured in the range of the calibration curve, because the samples were pre-diluted more than six times with the extraction solution. The precision and accuracy of the assay were expressed as the RSD (%) and the relative error (RE) (%), respectively, and the lower limit of quantification (LLOQ) was within ± 20%, and the other QC levels were all within ± 15% for the nominal analytes (Table 1). The precision and accuracy of the assay were therefore consistent with European Medicines Agency (EMA) guidelines^23^. Regarding sample stability, the average concentrations at each level under the tested conditions were within ± 15% of the nominal concentration, (Supplementary Tables 2, 3) which was acceptable according to EMA^23^.

**Table 1:** Precision and accuracy for the quantification of *C*-Man-Trp (CMW) (n = 5).

Nominal Conc. (nM)	Mean Conc. (nM)	Intra-run		Inter-run	
		Precision (RSD, %)	Accuracy RE (%)	Precision (RSD, %)	Accuracy RE (%)
**< 300 nM of CMW (nM)**					
5	5.64	10.62	19.48	18.34	12.84
10	9.99	8.55	5.27	14.17	− 0.08
25	24.59	6.92	− 2.92	7.98	− 1.63
50	48.70	2.76	− 0.45	5.63	− 2.60
**≥ 300 nM of CMW (nM)**					
5	5.22	9.22	− 2.43	19.86	4.45
10	10.45	11.53	− 3.01	12.71	4.49
25	23.52	7.46	− 9.56	10.95	− 5.94
50	48.36	4.75	− 11.87	7.75	− 3.29

**Figure 1:**
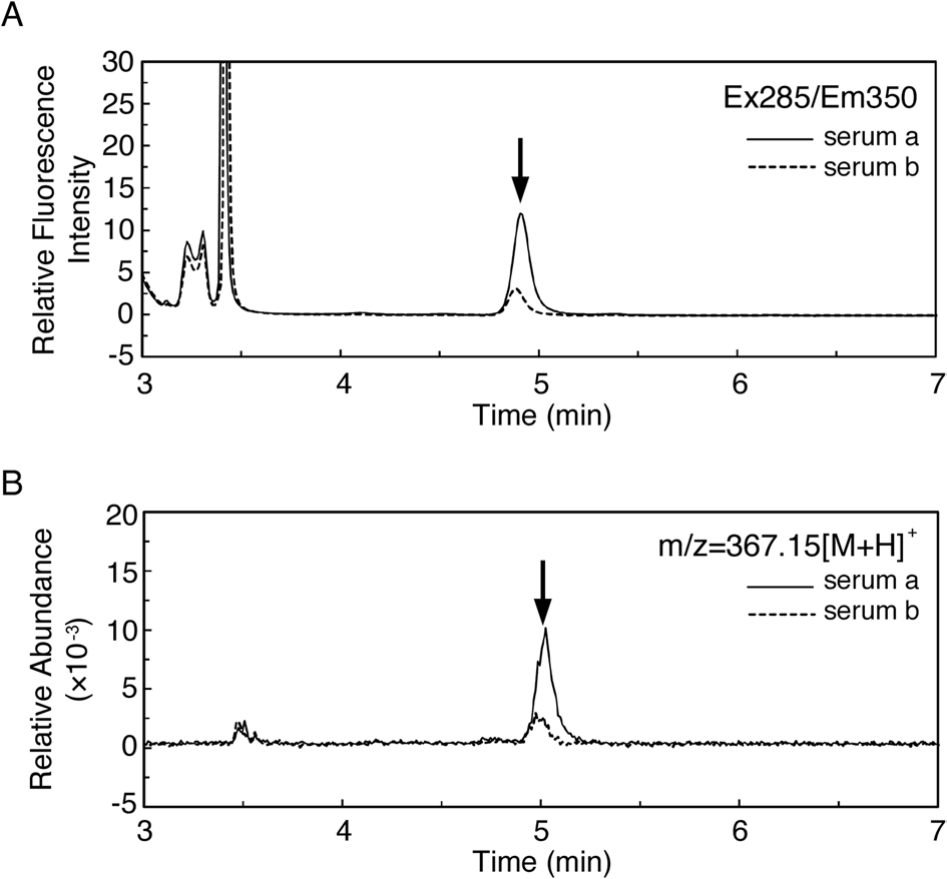
Measurement of *C*-mannosyl tryptophan (CMW) in blood samples from type 2 diabetic patients with or without renal dysfunction. Serum samples were prepared by organic solvent extraction, followed by centrifugation, and analyzed by UPLC. CMW level was quantified by measuring (**A**) the fluorescence intensity (excitation at 285 nm/emission at 350 nm). The identity of CMW was confirmed by measuring (**B**) the mass abundance (m/z value of 367.15 [M + H]^+^). Sera **a** (thin line) and **b** (dotted line) are from the type 2 diabetic patients with and without renal dysfunction, respectively. Arrows indicate the peak corresponding to CMW.

The typical elution patterns of CMW are shown in Fig. 1. CMW was detected by monitoring the fluorescence intensity (excitation at 285 nm/emission at 350 nm) (Fig. 1A) and mass abundance (Fig. 1B) of serum samples. CMW was detected on the basis of the main fluorescence intensity peak at 4.9 min (Fig. 1A, arrow) and the main mass-abundance peak at 5.0 min (Fig. 1B, arrow). The mass of the target peak was used to confirm CMW (m/z value of 367.15 [M + H]^+^). Target peaks were further confirmed as CMW by adding synthesized CMW to the samples. Serum **a** was from a T2D patient with renal dysfunction and serum **b** sample was from a T2D patient without renal dysfunction. The level of CMW was quantified in the samples on the basis of the calibration curve constructed from synthesized CMW, as described previously^22^. The level of CMW was higher in serum **a** than in serum **b**.

### Clinical characteristics and regression analysis for serum CMW

**Figure 2:**
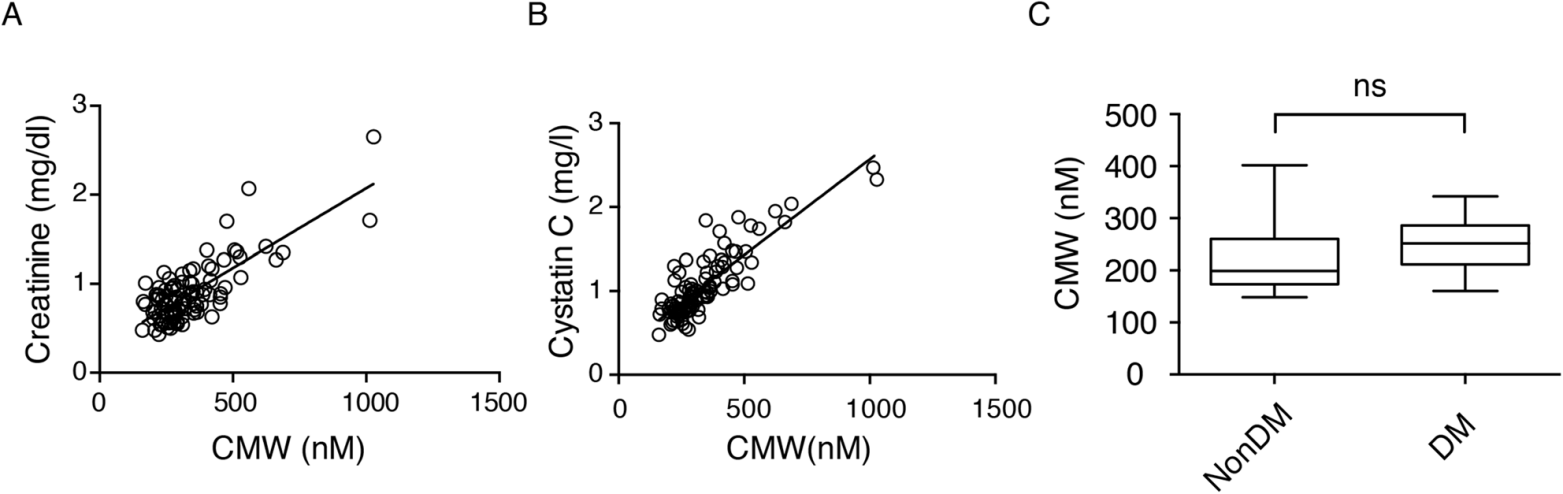
(**A**,**B**) Correlation of serum *C*-mannosyl tryptophan (CMW) with (**A**) serum creatinine (r = 0.769, P < 0.001) and (**B**) cystatin C (r = 0.854, P < 0.001) in the patients with type 2 diabetes (n = 99). (**C**) Serum CMW of the subjects without diabetes or renal dysfunction (CMW; 221 ± 16.7 nM, n = 18) and age-matched diabetic patients whose eGFR > 60 ml/min/1.73 m^2^ (CMW, 248 ± 10.3 nM; duration of diabetes, 10.8 ± 1.8 years; n = 23). ns; not significant.

The clinical characteristics and factors associated with serum CMW level assessed by univariate regression analysis are shown in Table 2. Age, duration of diabetes, treatment for diabetes, serum creatinine level, eGFR, and urine albumin-to-creatinine ratio (ACR) were significantly correlated with serum CMW level (Table 2, Fig. 2A). Among age, treatment, serum creatinine level, and ACR, multivariate regression analysis revealed the significant correlation of serum creatinine level with serum CMW level, with an adjusted *r*^2^ of 0.59 (P < 0.001). By confirming the association between renal function and serum CMW level, cystatin C level was significantly associated with serum CMW level (Fig. 2B). Serum CMW level did not significantly differ between the diabetic patients without renal dysfunction and the nondiabetic control subjects (Fig. 2C).

**Table 2:** Correlation between serum CMW level and clinical characteristics (n = 99).

	Mean	SE	*r*	P value
Sex (% women)	46.4	5.0	0.09224	0.3638
Age (y)	68.4	1.2	0.2412	0.0162
Duration of diabetes (y)	17.1	1.0	0.3148	0.0015
BMI (kg/m^2^)	24.6	0.4	0.0275	0.787
Fasting plasma glucose (mg/dl)	143.1	3.3	0.1119	0.2702
HbA1c (%)	7.5	0.1	− 0.1094	0.2809
Systolic BP (mmHg)	137.8	1.7	0.05711	0.5745
Diastolic BP (mmHg)	73.9	1.3	− 0.01834	0.857
Treatment (diet/OHA/I)^a^	2.3	0.1	0.2062	0.0406
Creatinine (mg/dl)	0.9	0.03	0.7688	< 0.0001
eGFR (ml/min/1.73 m^2^)	65.6	2.0	− 0.6859	< 0.0001
ACR (mg/g)	115.4	31.5	0.3129	0.0016
CMW (nM)	336.2	14.6	NA	NA

*eGFR*, estimated glomerular filtration rate; *ACR*, urine albumin-to-creatinine ratio; *CMW*, *C*-mannosyl tryptophan.

^a^Treatment: Diet, 1; Oral Hypoglycemic Agents, 2; Insulin, 3.

### Assessment of renal function

**Figure 3:**
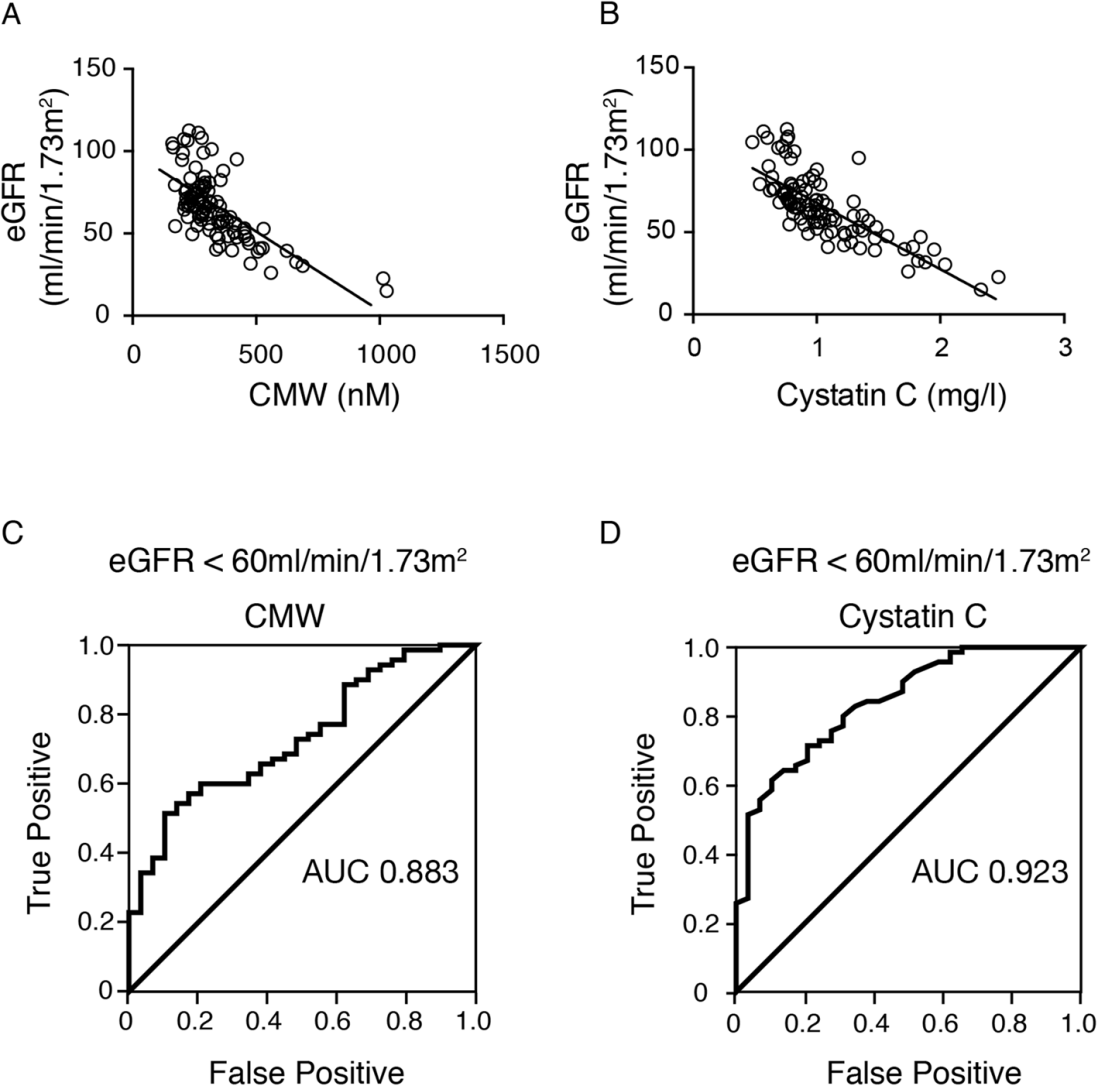
(**A**,**B**) Correlation of eGFR and (**A**) serum *C*-mannosyl tryptophan (CMW) (r = − 0.686, P < 0.001) and (**B**) cystatin C (r = − 0.770, P < 0.001) (n = 99). (**C**,**D**) ROC of (**C**) serum CMW and (**D**) cystatin C to detect eGFR < 60 ml/min/1.73 m^2^.

Based on the significant correlation of serum CMW with the markers of renal function, we further validated serum CMW as a biomarker for renal function by comparing it with serum cystatin C. Both serum CMW and cystatin C levels were significantly associated with eGFR (Fig. 3A,B). The receiver operating characteristic (ROC) curve used to detect eGFR < 60 ml/min/1.73 m^2^ revealed that the cutoff serum CMW level was 337.5 nM (area under curve [AUC], 0.883; specificity, 0.88; sensitivity, 0.82) (Fig. 3C), while the cutoff cystatin C level was 1.06 mg/l (AUC, 0.923; specificity, 0.93; sensitivity, 0.79) (Fig. 3D). There was no significant difference between these AUCs.

### Assessment of vascular complications

**Figure 4:**
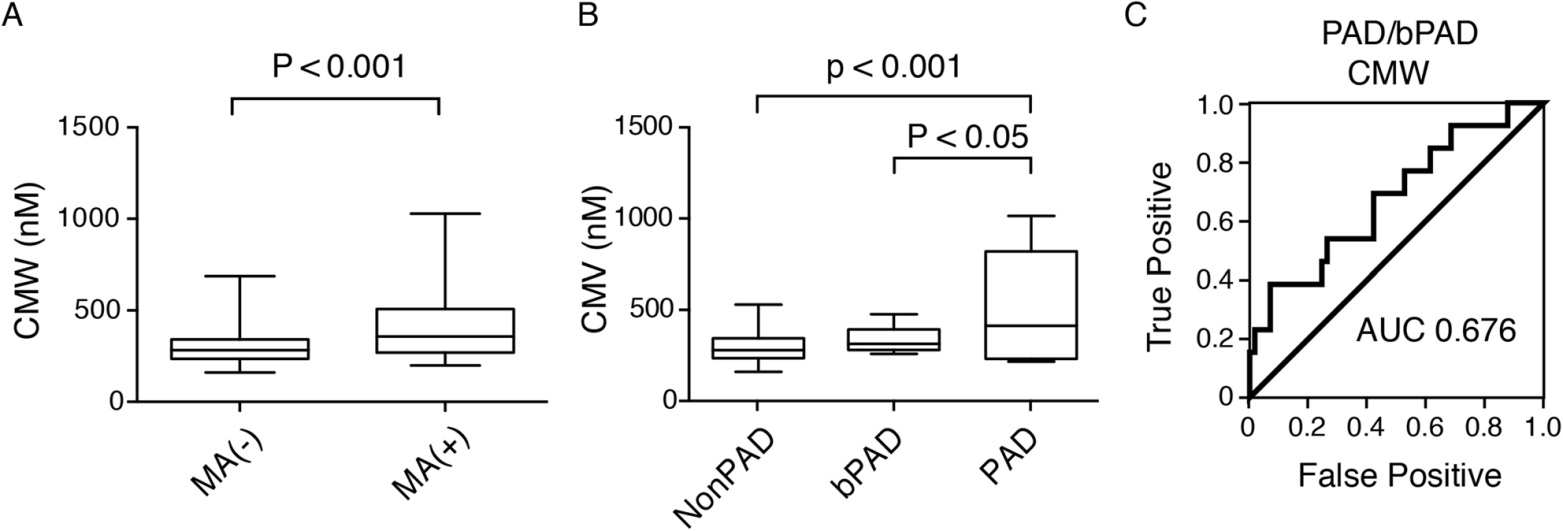
(**A**) *C*-Mannosyl tryptophan (CMW) levels in type 2 diabetic patients with the past history of macroangiopathy (MA) (n = 30) and those without it (n = 69). (**B**) Serum CMW levels in type 2 diabetic patients with peripheral artery disease (PAD) (n = 5), borderline PAD (bPAD) (n = 8), and without PAD (NonPAD) (n = 57). (**C**) ROC curve of serum CMW to detect PAD/bPAD.

Since eGFR and cystatin C levels are known to be associated with several vascular complications^24^, we hypothesized that CMW could be clinically more useful for assessment of vascular complications in T2D. As shown in Fig. 4A, serum CMW level was significantly higher in the group with past histories of macrovascular complications. Next, we analyzed which factors regarding vascular complications correlate with serum CMW level. The severity of retinopathy, ankle-brachial pressure index (ABI), brachial-ankle pulse wave velocity (PWV), and previous event(s) of macrovascular complications were correlated with serum CMW levels (Table 3). Interestingly, among the biomarkers for renal function, only serum CMW level correlated with ABI. To investigate the association between serum CMW level and the peripheral arterial disease (PAD) in more detail, we divided the patients into three groups: PAD (ABI < 0.9), borderline PAD (bPAD, 0.9 ≦ ABI < 1) and nonPAD (1 ≦ ABI). Previous reports revealed the cumulative risk of cardiovascular disease in patients with bPAD^25^. As shown in Fig. 4B, the PAD group showed significantly higher serum CMW levels than the bPAD or nonPAD group. The ROC curve used to detect PAD/bPAD revealed that the cutoff serum CMW level was 408.6 nM (AUC, 0.676; specificity, 0.93; sensitivity, 0.38) (Fig. 4C).

**Table 3:** Comparisons of correlation between markers of renal function and vascular complications of diabetes.

	Mean	SE	CMW		Creatinine		Cystatin C		eGFR		n
			*r*	P value	*r*	P value	*r*	P value	*r*	P value	
Retinopathy^a^	1.54	0.08	0.293	0.0048	0.3303	0.0014	0.3576	0.0005	− 0.2594	0.013	91
ABI	1.07	0.01	− 0.2916	0.0143	− 0.2174	0.0706	− 0.2288	0.0568	0.1549	0.2004	70
PWV	1877.4	44.5	0.2476	0.0388	0.4008	0.0006	− 0.3967	0.0007	0.2603	0.0295	70
MAs^b^	0.303	0.05	0.367	0.0018	0.4672	< 0.0001	0.3223	0.0065	− 0.3777	0.0013	99

*ABI*, ankle-brachial pressure index; *PWV*, Brachial-ankle pulse wave velocity; *CMW*, *C*-mannosyl tryptophan.

^a^Retinopathy: Nonretinopathy, 1; Nonproliferative retinopathy, 2; Proliferative retinopathy, 3.

^b^MAs: Previous event(s) of macrovascular complications after the diagnosis of type 2 diabetes; Yes, 1; No, 0.

## Discussion

Although CMW is a promising candidate biomarker of renal function, the accuracy of quantification and characterization in diabetic patients are challenging yet pivotal for its clinical use. In this study, we first accurately quantified serum CMW using the novel assay based on the measurement of the fluorescence intensity of CMW by HILIC. We then characterized serum CMW levels in patients with T2D.

Several metabolites including CMW have been suggested as candidates for evaluating renal function, but the limited reports revealed the absolute and accurate quantification of those metabolites. Furthermore, a controversial study excluded CMW from these candidate metabolites for the evaluation of renal function^15^. To prevent the progression of diabetic nephropathy, staging based on accurate assessment of renal function is essential to initiate or adjust the treatment in patients with diabetes. In the current study, we first defined the cutoff serum CMW level for eGFR < 60 ml/min/1.73 m^2^ on the basis of the novel assay results for the patients with T2D. We selected eGFR < 60 ml/min/1.73 m^2^ as it is defined as G3a category by Kidney Disease Improving Global Outcome (KDIGO) CKD guideline 2012. By using this value, we confirmed that the value correlates well with serum creatinine and cystatin C level, which is independent of fasting blood glucose levels. Which factors of renal function could correlate with serum CMW as a single metabolite were not previously fully investigated. Our findings revealed closer correlation of CMW level with glomerular filtration factors than with excretion. Thus, even in cases in which several factors affect the conventional biomarkers, such as serum creatinine or cystatin C levels, serum CMW level could be clinically useful for assessing glomerular filtration function.

*C*-Mannosylation is a post-translational modification of secretory or membrane proteins^5^. Although it requires clarification, monomeric CMW in blood could be generated in part by the degradation of *C*-mannosylated proteins via ubiquitin–proteasome^26^, ER-associated degradation (ERAD)^27^, the autophagic lysosomal pathway^28^, and/or extracellular proteases^29^ in cells. Interestingly, autophagy induction was reported to play a protective role against tissue damage in the kidney injury^30^. It may therefore be compatible with a pathophysiological response in renal damage, whereby the CMW level is upregulated in the blood of patients with renal dysfunction through the upregulated autophagy in damaged renal tissues. Further investigation is required to clarify the relevant connection between CMW and autophagy-related diseases, such as kidney injury.

This is the first study to show that serum CMW level negatively correlates with ABI. Previous reports revealed the association between ABI and cystatin C level or eGFR determined on the basis of serum creatinine level^24^. Our findings indicate a closer association of serum CMW level with ABI than with cystatin C. Furthermore, serum CMW levels were increased with the progression from borderline PAD to PAD, which suggests that serum CMW could be a biomarker for not only assessing the onset of PAD, but also for monitoring the progression of PAD in daily clinical practice. Considering the time and cost to measure ABI frequently in high-risk patients with T2D, it could be less expensive and easier to quantify CMW levels from the serum sample to assess the onset and progression of PAD.

In our previous study, protein *C*-mannosylation was increased in the aortic vessels of the T2D model using diabetic Zucker fatty rats^31^. The level of *C*-mannosylated thrombospondin1 (TSP1) was increased in the aortic tissues of diabetic Zucker rats, but we did not investigate the level of blood CMW. TSP1, a *C*-mannosylated protein^32^, functions at the cell surface and in the extracellular matrix to regulate cellular interactions and signaling via binding to integral molecules, such as TGF-β, integrins, collagens, proteoglycans, CD47, CD36, and calreticulin^33,34^. In diabetes, TSP1 is involved in various diabetic complications through the activation of TGF-β signaling^35^. Increased *C*-mannosylated TSP1 levels might therefore play a causative role in TGF-β-related pathological processes in the damaged aortic vascular tissues in T2D. Taken together, these findings suggest that upregulation of blood CMW might be involved in the pathogenesis of vascular complications in diabetes.

In summary, we first quantified the serum CMW levels in patients with T2D by using our recently developed novel assay. We then characterized them in relation to the clinical factors, which revealed that CMW level was associated with the levels of markers of renal function, especially glomerular filtration, independent of fasting glucose levels. Finally, we demonstrated that the value of serum CMW level was increased in the patients with T2D with borderline PAD or PAD. Serum CMW level determined by our novel assay could be a promising biomarker for the onset or progression of PAD as well as for renal glomerular function in patients with T2D.

## Materials and methods

### Study design

This single-center cross-sectional design study was carried out in accordance with the Declaration of Helsinki. The study protocol was approved by the Wakayama Medical University Ethics Committee (#1825, #2343). Written informed consent was obtained from all participants in this study.

### Study population

We enrolled 121 patients with T2D who were diagnosed as type 2 diabetes and observed at the outpatient clinic at Wakayama Medical University Hospital between October 2018 and May 2020. T2D was diagnosed based on the criteria set by the Japan Diabetes Society^14^. Among enrolled patients, we analyzed 99 patients (46 women; age, 68.4 ± 1.2 years; duration of diabetes, 17.1 ± 1.0 years), who were able to be evaluated and who had the required clinical data, including renal function, and did not meet any of the exclusion criteria described below. Fifteen subjects without diabetes or renal dysfunction were analyzed as controls (10 women; age, 49 ± 3.2 years). We excluded patients who had endocrine, hepatic or apparent renal disorders other than diabetic kidney disease, and those who were otherwise judged to be ineligible by the attending physician. All participants were interviewed about their general condition, including fever, and any recent infections.

### Synthesis of C2-α-*C*-mannosyl-l-tryptophan (CMW)

C2-α-*C*-Mannosyl-l-tryptophan (CMW) was synthesized as previously described^36^. The purity and identity of the final product were verified by ^1^H NMR spectroscopy and matrix-assisted laser desorption ionization (MALDI) mass spectrometry. The proton chemical shifts and coupling constants were consistent with those previously reported, and the mass on MALDI mass spectrometry was consistent with the expected mass of the correct product.

### Assessment of serum CMW

Blood samples were collected and serum fractions were prepared by conventional methods. The samples were frozen and stored at − 80 °C until use. To measure CMW, the serum samples were thawed in cold water, and a 25-μl aliquot of the serum was mixed with 125 μl of extraction solution (methanol:acetonitrile:formic acid = 50:49.9:0.1), and centrifuged at 12,000 × *g* for 15 min at 4 °C. Then the supernatant of 140 μl was further filtered using a 0.20-μm polytetrafluoroethylene (PTFE) syringe filter. The CMW sample (5 μl) was analyzed and quantified by a UPLC H-Class system with BEH amide column (Waters Corporation) as previously described^22^. CMW was quantified using chemically synthesized CMW as a standard molecule, by measuring the fluorescence (excitation at 285 nm/emission at 350 nm). CMW levels in the serum aliquots were comparable with each other. The identity of CMW was alternatively confirmed by measuring mass abundance (m/z value of 367.15 [M + H]^+^ for CMW) in randomly-selected cases. Empower 3 software was used to collect and process data.

### Analytical method validation

The validity of the CMW assay method was assessed for linearity, sensitivity, specificity, recovery, accuracy, precision, and stability in accordance with the guideline on bioanalytical method validation of EMA^23^.

The linearity was assessed for each analyte by statistical analysis of the calibration curves by dilution of the standard stock solution of CMW with an extraction solution to prepare seven different concentrations (5–200 nM).

Regarding sensitivity, LOD and LOQ of the methods were determined by applying a series of diluted CMW based on signal-to-noise (S/N) approach^37^. The S/N ratio of three is acceptable for estimating LOD. LLOQ was defined as the lowest analyte concentration which can be reliably quantified with a S/N ratio of at least ≥ 5.

The sample extraction recovery was determined by spiking aliquots of four different serum samples with four QC concentrations (5 nM [LLOQ], 10 nM [Low quality control/LQC], 25 nM [Middle quality control/MQC], and 50 nM [High quality control/HQC]) of CMW. The CMW level was measured in two sample aliquots prepared by spiking with CMW pre-extraction (A) or post-extraction (B). The endogenous level of CMW was measured in each non-spiked sample aliquot (C). Extraction recovery of CMW was calculated as: Extraction recovery (%) = (A − C)/nominal QC concentration × 100. Matrix effects were investigated using four QC concentrations of CMW in different serum samples. Matrix effect of CMW was calculated as: Matrix effect (%) = (B − C)/nominal QC concentration × 100. The relative standard deviation (% RSD) calculated from six serum samples for CMW should not be > 20%, according to the EMA guidelines^23^.

Accuracy and precision were assessed by analysis of serum samples spiked with LLOQ, LQC, MQC, and HQC accompanied with a set of calibration standard curves in each case. Five different serum samples at four QC concentrations were prepared as one batch and were analyzed to assess intra-run variation. The four batches of QC samples were analyzed on different days to assess inter-run variation. The intra- and inter-run precisions (% RSD) and accuracies (relative error, % RE) of LLOQ should be within 20%, and the three QC levels should be within 15%.

Sample stability was assessed using three different serum samples spiked with CMW with different concentrations (LQC and HQC) under the following conditions: (1) short-term stability of CMW is serum samples processed at 4 °C for 8 h, or stored in the auto-sampler at 8 °C for 8–24 h after extraction, (2) long-term stability of CMW is serum samples processed at − 80 °C for 3 weeks, (3) freeze and thaw stability of CMW is serum samples from freezer storage conditions to 4 °C (three cycles with at least 24-h intervals). For stability, the CMW level should be within ± 15% of the nominal QC concentration under different given conditions.

### Laboratory measurements

Laboratory measurements including hemoglobin A1c (HbA1c) were assessed at Wakayama Medical University using routine laboratory methods. The estimated glomerular filtration rate (eGFR) was calculated using the following equation, specific to the Japanese population: eGFR (ml/min/1.73 m^2^) = 194 × (age [years]) − 0.287 × (serum creatinine [Cr; mg/dl]) − 1.904 (× 0.739 for women). The urinary albumin‐to‐creatinine ratio (ACR) was then expressed in milligrams per gram of Cr (mg/g).

### Assessments of vascular complications

Measurement of the ABI and brachial-ankle PWV were according to a standard protocol using BP-203RPEIII (Fukuda Colin, Tokyo, Japan) as described previously^38^. Briefly, patients had cuffs placed on both arms and both ankles in the supine position and were then rested for 5 min. The systolic pressures of the bilateral brachial, posterior tibial, and dorsalis pedis arteries were measured simultaneously. The ABI values of both legs were calculated by dividing the maximum systolic pressure in the right and left ankles by the higher of the two brachial systolic pressures. The lower ABI value was employed as a diagnosis of PAD or borderline PAD (bPAD); PAD and borderline PAD were defined as an ABI < 0.9 and 0.9 ≦ ABI < 1, respectively^39–41^. Among 99 patients, ABI and PWV were analyzed in 70 patients based on the clinical requirement (31 women; age, 68.1 ± 1.5 years; duration of diabetes, 16.8 ± 1.2 years). In this cohort, concentrations of CMW for each severity of PAD (PAD, bPAD, without PAD), ROC curve of serum CMW to detect PAD/bPAD were analyzed as shown in Fig. 4. Grading of retinopathy was based on international clinical diabetic retinopathy and diabetic macular edema disease severity scales^42^. A medical retina specialist, masked to all other subject information, graded the diabetic retinopathy, as previously reported^43^.

### Statistical analysis

Student’s t test or one-way ANOVA followed by post hoc Tukey’s test were applied to determine statistical difference between two groups or between more than two groups, respectively, unless otherwise noted. Associations between serum CMW and clinical characteristics were analyzed by single linear and multivariate regression model. To evaluate the absolute serum CMW levels to detect renal dysfunction, we first constructed ROC curves and determined their area under the curve (AUC), then we calculated the AUC of cystatin C. We compared the AUC of the two ROC curves as previously reported^44^. All statistical analyses were performed with GraphPad Prism version 6.00 (GraphPad Software Inc, San Diego, U.S.A.) or JMP Pro14 (SAS Institute Inc., Cary, NC, U.S.A.). P-value < 0.05 was considered to be statistically significant.

## Supplementary Information

Supplementary Tables.
