# Supplementary material for: Quantification of serum C-mannosyl tryptophan by novel assay to evaluate renal function and vascular complications in patients with type 2 diabetes
Source: Sci Rep. 2021 Jan 21;11:1946. doi: 10.1038/s41598-021-81479-y (PMC7820242; doi:10.1038/s41598-021-81479-y)
Supplement: Supplementary file 1 — Supplementary Tables. [file 41598_2021_81479_MOESM1_ESM.docx]

**Supplementary Information**

**Quantification of serum *C*-mannosyl tryptophan by novel assay to evaluate renal function and vascular complications in patients with type 2 diabetes**

Shuhei Morita^1,*^, Yoko Inai^2^, Shiho Minakata^2^, Shohei Kishimoto^1^, Shino Manabe^3,4^, Naoyuki Iwahashi^5^, Kazuhiko Ino^5^, Yukishige Ito^6,7^, Takashi Akamizu^1^, and Yoshito Ihara^2,*^

^1^First Department of Medicine, ^2^Department of Biochemistry, ^5^Department of Obstetrics and Gynecology, Wakayama Medical University, 811-1 Kimiidera, Wakayama, Wakayama 641-0012, Japan

^3^Pharmaceutical Department & The Institute of Medicinal Chemistry, Hoshi University, 2-4-41 Ebara, Shinagawa, Tokyo 142-8501, Japan

^4^Research Center for Pharmaceutical Development, Graduate School of Pharmaceutical Sciences & Faculty of Pharmaceutical Sciences, Tohoku University, 6-3 Aoba, Aramaki, Sendai, Miyagi, 980-8578, Japan

^6^RIKEN Cluster for Pioneering Research, 2-1 Hirosawa, Wako, Saitama 351-0198, Japan

^7^ Department of Chemistry, Graduate School of Science, Osaka University, 1-1 Machikaneyama, Toyonaka, Osaka 560-0043, Japan

*Corresponding author:

Shuhei Morita^1^ and Yoshito Ihara^2^

^1^First Department of Medicine, Wakayama Medical University

811-1 Kimiidera, Wakayama, 641-0012, Japan

Phone/Fax: +81-73-441-0625/+81-73-445-9436

e-mail: smorita@wakayama-med.ac.jp

^2^Department of Biochemistry, Wakayama Medical University

e-mail: y-ihara@wakayama-med.ac.jp

**Supplementary Data**

Supplementary Table 1. Comparison of recovery efficiency of CMW for the quantification (n = 5)

| Nominal Conc. (nM) | Absolute recovery (%) | Matrix effect (%) |
| --- | --- | --- |
| 5 nM  10 nM  25 nM  50 nM | 105.26 ± 16.13  88.36 ± 12.11  94.09 ± 4.76  88.17 ± 4.50 | 117.47 ± 10.59  106.30 ± 10.87  106.00 ± 3.62  100.12 ± 3.62 |

Supplementary Table 2. Stability of CMW in serum in various conditions (n = 4)

|  | 4°C, 8 h | | -80°C, 3 weeks | | Three freeze-thaw cycles | |
| --- | --- | --- | --- | --- | --- | --- |
| Nominal Conc. (nM) | Mean Conc.  (nM) | RE (%) | Mean Conc.  (nM) | RE (%) | Mean Conc.  (nM) | RE (%) |
| 10 nM  50 nM | 8.58  48.38 | -14.19  -3.24 | 9.74  44.38 | -2.57  -11.24 | 9.75  46.90 | -2.48  -6.21 |

Supplementary Table 3. Stability of CMW in serum, stored in the auto-samples at 8°C (n = 3)

|  | 8 h | | 16 h | | 24 h | |
| --- | --- | --- | --- | --- | --- | --- |
| Nominal Conc. (nM) | Mean Conc.  (nM) | RE (%) | Mean Conc.  (nM) | RE (%) | Mean Conc.  (nM) | RE (%) |
| 10 nM  50 nM | 9.89  48.43 | -1.07  -3.14 | 11.07  49.98 | 10.73  -0.04 | 10.52  47.53 | 5.16  -4.94 |

CMW, *C*-Mannosyl tryptophan
